# Hypoglycemia Prevention Practice and Associated Factors among Diabetic Patients on Follow-Up at Public Hospitals of Central Zone, Tigray, Ethiopia, 2018

**DOI:** 10.1155/2020/8743531

**Published:** 2020-03-13

**Authors:** Gebrewahd Bezabh Gebremichael, Teklewoini Mariye Zemicheal

**Affiliations:** ^1^School of Nursing College of Health Sciences Mekelle University, Tigray, Ethiopia; ^2^School of Nursing College of Health Sciences Axum University, Tigray, Ethiopia

## Abstract

**Background:**

Hypoglycemia is an acute medical situation that occurs when blood glucose level falls below 70 mg/dl. Although prevention of hypoglycemia is one cornerstone in the management of diabetes mellitus, its prevention practice among patients with diabetes mellitus is insufficiently studied. Moreover, the existed scarce literature in Ethiopia revealed hypoglycemia prevention practice is inadequate. Thus, this study tried to assess hypoglycemia prevention practices and associated factors among diabetic patients.

**Methods:**

Hospital-based cross-sectional study design was employed from March 1 to April 1, 2018, in the central zone of Tigray regional state of Ethiopia. A total of 272 diabetes mellitus patients selected by a systematic random sampling method were included in the study. Data were entered into Epi-data version 3.1 and exported to SPSS version 23 for further analysis. The binary logistic regression model (AOR, 95% CI, and *p* value < 0.05) was used to determine the predictors of hypoglycemia prevention practice.

**Results:**

The mean age of respondents was 43.62 years, and about 100 (63.2%) participants had good hypoglycemia prevention practice. Good knowledge on hypoglycemia (AOR = 10.34; 95% CI [5.41, 19.89]), having a glucometer at home (AOR = 3.02; 95% CI [1.12, 8.12]), favorable attitude towards diabetes mellitus (AOR = 2.36 CI [1.26, 4.39]), and being governmental employee (AOR = 5.19, 95% CI [1.63, 16.58]) were positive predictors of good hypoglycemia prevention practice. However, being divorced (AOR = 0.13, 95% CI [0.32, 0.53]) was found negatively associated with good hypoglycemia prevention practice.

**Conclusion:**

Only two-thirds of the study participants were found to have good hypoglycemia prevention practices. Healthcare personnel and Ethiopian diabetic association should promote patients' attitude towards DM and knowledge on hypoglycemia by strengthening information, education, and communication program. Stakeholders should also try to provide glucometers to diabetic patients.

## 1. Background

Hypoglycemia, defined as a blood glucose level <70 mg/dL (3.9 mmol/L), is the most common and highly feared acute complication of diabetes mellitus, particularly in patients with insulin therapy. Hypoglycemia is the major limiting factor in the glycemic management of type 1 and type 2 [1]. Although there is a lack of real-world data on hypoglycemia, a recent analysis of the existing literature has shown that hypoglycemia is more common in clinical practice [2].

Nonsevere hypoglycemia rates in type 1 diabetes mellitus ranges from 91.0 and 136.8 episodes per person per year, and severe hypoglycemia rates ranged from 0.7 to 1.59 episodes per person per year in the real world. In type 2 diabetes mellitus, nonsevere rates of hypoglycemia ranged from 0.224–35.3 episodes per person per year and severe hypoglycemia rates ranged from 0.00 to 0.12 episodes per person per year in the real-world studies [2].

In addition to causing distress, severe hypoglycemia is associated with major vascular events such as stroke, myocardial infarction, acute cardiac failure, ventricular arrhythmias, and sudden death. Complications of hypoglycemia are also associated with short-term disability and higher healthcare costs [3]. An observational trial showed a 3.4 times higher mortality among patients who reported severe hypoglycemia at baseline [4]. Consequently, clinical decisions on glycemic control are often made according to the relative benefits of insulin therapy versus the risk of hypoglycemia [5].

Individuals at risk for hypoglycemia should be asked about symptomatic and asymptomatic hypoglycemia at each encounter. Glucose (15–20 g) is the preferred treatment for the conscious individual with blood glucose <70 mg/dL, although any form of carbohydrate that contains glucose may be used. Fats and carbohydrate sources which are high in protein should not be used to treat or prevent hypoglycemia. Glucagon should be prescribed for all individuals with blood glucose <54 mg/dL. Glucagon is indicated for the treatment of hypoglycemia in people unable or unwilling to consume carbohydrates by mouth. Caregivers, school personnel, or family members of these individuals should know where it is and when and how to administer. Once the glucose returns to normal, the individual should be counseled to eat a meal or snack to prevent recurrent hypoglycemia [6].

Hypoglycemia prevention is a critical component of diabetes management. Self-monitoring of blood glucose (SMBG) and continuous glucose monitoring (CGM) (recurrent asymptomatic hypoglycemia) are essential tools to assess therapy and detect incipient hypoglycemia. Patients should understand situations that increase their risk of hypoglycemia, such as when fasting for tests or procedures, when meals are delayed, during and after the consumption of alcohol, during and after intense exercise, and during sleep [3, 6].

Nevertheless, hypoglycemia might be a major challenge in Africa, which has the greatest predicted increase in both the burden of diabetes and associated diabetic complications but yet contributes the lowest in the global annual healthcare expenses with regard to diabetes care [7, 8]. Hypoglycemia is a significant contributor to total diabetes mellitus expenditure. The health economic burden associated with hypoglycemia can be related with extensive economic loss in diabetes mellitus patients with their family, to health systems and to the national economies through direct and indirect costs [9, 10].

Besides, hypoglycemia has shown negative impact on quality of life and productivity in the workplace [11]. However, because of lack of comprehensive data, particularly in developing countries, the economic impact of hypoglycemia on patients and healthcare providers in developing countries has been remained poorly understood.

Enforcement of hypoglycemia prevention practices in the management of diabetes mellitus and identifying the associated factors with hypoglycemia prevention practices such as optimization of insulin treatment, improved access to blood glucose testing, patient education and developing good knowledge on hypoglycemia, and promoting favorable attitude towards diabetes mellitus may help patients more effectively to delay the onset and reduce the development of complication that leads to a prolonged hospitalization and reduced quality of life [12, 13].

However, except a single study done in Amhara Region which indicated high magnitude of poor hypoglycemia prevention practices [14, 15], hypoglycemia prevention practices among patients with diabetes mellitus is insufficiently studied in Tigray Region. Thus, this study tried to assess hypoglycemia prevention practices and associated factors among patients with diabetes mellitus in public hospitals of the central zone of Tigray, Ethiopia.

## 2. Methods

### 2.1. Study Area and Period

The study was conducted in four public hospitals (Aksum University Referral Hospital, Saint Marry Hospital, Adwa Hospital, and Abyi Adi Hospital) of the central zone, from March 1 to April 1, 2018. These hospitals provided a follow-up service for a total of 1600 diabetic patients.

### 2.2. Study Design

Hospital-based cross-sectional study design was carried out among diabetes mellitus patients.

### 2.3. Population

The source population was all diabetic mellitus patients who were on follow-up at the public hospitals of the central zone of Tigray during the data collection period, and the study population was all the sampled diabetes mellitus patients.

### 2.4. Sample Size Determination

Sample size was determined using a single population proportion formula using EPI Info software version 7.1.1 considering 95% confidence interval, 5% marginal error (d), and 21.4% prevalence (P) of good hypoglycemia prevention practice taken from a study conducted in South Gondar, Northwest Ethiopia [14]. By adding 5% nonresponse rate, the final sample size was 272.

### 2.5. Sampling Procedure and Techniques

The sample size was proportionally allocated into each hospital based on the number of diabetic patients on follow-up in each of them. Then, sampling interval was calculated by dividing the total number of diabetic patients in the selected hospitals to the required sample size (*K* = *N*/*n*). Participants were selected by systematic random sampling every *K*^th^ interval. However, the first individual participant was selected using simple random sampling technique by the lottery method.

### 2.6. Variables

Dependent variables: hypoglycemia prevention practice.

Independent variables: sociodemographic (sex, age, residence, marital status, educational status, and occupation), clinical characteristics (type of DM, diabetic complication, family history of DM, attending diabetic education, being member of diabetic association, having a glucometer, and knowing glucose level), attitude towards DM, and knowledge towards hypoglycemia.

### 2.7. Data Collection Tool and Technique

The data collection tool was adapted from similar literatures [14, 16, 17]. The questionnaire had three parts. Part I contained sociodemographic data. Part II included health related data, knowledge on hypoglycemia, and attitude towards diabetes mellitus of participants. Knowledge on hypoglycemia was assessed using 8 questions, and participants score ranges from 0 to 8. Attitude towards diabetes mellitus was assessed using 5 Likert scale questions. Part III contained hypoglycemia prevention practices. Hypoglycemia prevention practice was determined using 11 questions with only one possible answer. Data were collected through a face to face interview using the structured questionnaire by four bachelor of science nurses with two supervisors.

### 2.8. Data Quality Assurance

The questionnaire was initially prepared in English and translated into the local language (Tigrigna) by an individual who has a good ability of the two languages. The questionnaire was translated back to English by the different person to ensure consistency. Two-day training was given for data collectors and supervisors. A week prior to the actual data collection period, the questionnaire was pretested on 5% of the total sample size. The collected data were checked at the spot and cleaned in SPSS version 22.0.

### 2.9. Data Processing and Analysis

Data were coded and entered into Epi-Data version 3.1, and then it was exported to SPSS version 22.0 for further analysis. The binary logistic regression model was used to identify the predictors of hypoglycemia prevention practice. Variables which were potential independent predictors on bivariate analysis with *p* value ≤ 0.2 were entered to multivariable logistic regression analysis. Finally, the magnitude of association between the dependent and independent variables was measured using odds ratios AOR with 95% confidence interval, and statistical significance was declared at *p* value < 0.05.

### 2.10. Operational Definitions

*Good practice*: participants who scored mean and above from the hypoglycemia prevention practice questions.

*Poor practice*: participants who scored below the mean value from the hypoglycemia prevention practice questions [14]. The mean score was calculated out of 12 by adding each participant's score on the hypoglycemia prevention practice questions and dividing it to the total number of participants.

*Good knowledge*: mean and above knowledge score of participants from the knowledge questions on hypoglycemia.

*Poor knowledge*: less than mean knowledge score of participants from the knowledge questions on hypoglycemia. The mean knowledge score was calculated out of 7.

*Favorable attitude*: mean and above score of participants from the attitude questions towards diabetes mellitus.

*Unfavorable attitude*: less than mean score of participants from the attitude questions towards diabetes mellitus.

### 2.11. Ethical Consideration

Ethical clearance was obtained from the Institutional Review Board of Aksum University, College of Health Sciences. Permission was obtained from the medical directors of the respective public hospitals. Verbal consent was obtained from each of the study participants. Respondents were allowed to refuse or discontinue participation at any time they want. Information was recorded anonymously. Confidentiality and privacy were ensured throughout the study.

## 3. Results

### 3.1. Sociodemographic Profile of the Study Participants

A total of 272 diabetes mellitus patients were included in the study with a response rate of 100%. The mean age of the respondents was **43.62** ± **17.07**. One hundred forty-three (52.6%) were males. One hundred seventy-three (63.6%) of them were urban residents. One hundred thirty-eight (50.8%) participants did not had a formal education; two hundred sixteen (79.4%) respondents were Orthodox Christian followers, and 254 (93.4%) were Tegaru. Two hundred twenty-eight 228 (83.7%) of the respondents were unemployed. One hundred fifty-seven (57.7%) of respondents were married (Table 1).

### 3.2. Health Profile, Knowledge, and Attitude of the Study Participants

*The mean duration of diabetes mellitus of participants since diagnosis* was 4.61 with a minimum of one year and a maximum of 25 years. One hundred thirteen (41.5%) participants were with medically confirmed type I diabetes mellitus, and 58.5% were with type II diabetes mellitus. Forty-three (15.8%) participants had a family history of diabetes mellitus, and 49 (18%) respondents had a glucometer at home. One hundred seventy-nine respondents (65.8%) attended diabetic education. Seventy-seven of the total respondents (28.3%) had medically confirmed long-term diabetes mellitus complication. One hundred fifty-eight respondents (58.2%) knew their glucose level. Only fifty-seven (21%) participants were a member of the diabetic association, and 95 (34.9%) of participants did not know as there is diabetes mellitus association. One hundred seventy nine (65.8%) had good knowledge regarding hypoglycemia, and 132 (48.5%) had a favorable attitude regarding diabetes mellitus (Table 2).

### 3.3. Hypoglycemia Prevention Practice and Associated Factors

Nearly two-thirds, 63.2% (95% CI [58.6, 68.8]), of participants had good hypoglycemia prevention practice.

In the bivariate logistic regression analysis (sex, age, residence, marital status, educational status, and occupation), clinical characteristic type of DM, diabetic complication, family history of DM, attending diabetic education, having a glucometer, knowing glucose level, attitude towards DM, and knowledge towards hypoglycemia were found associated with hypoglycemia prevention practices. However, in the multivariable logistic regression analysis, only knowledge, favorable attitude, having glucometer, being governmental employee, and being divorced had shown statistically significant association with good hypoglycemia prevention practice.

Good knowledge on hypoglycemia was positively statically associated with good hypoglycemia prevention practice. The odds of having good knowledge was 10.34 (AOR = 10.34; 95% CI [5.41, 19.89]) times more strongly associated with good hypoglycemia prevention practice.

Having a glucometer at home showed a significant association with good hypoglycemia prevention practice. Participants who had glucometer at their home were found three (AOR = 3.02; 95% CI; 1.12–8.12) times more likely to have good hypoglycemia prevention practice than their counterparts.

Those who had favorable attitudes towards diabetes mellitus had more significant hypoglycemia prevention practice than participants who had unfavorable attitude. The odds of having favorable attitudes towards diabetic mellitus was 2.36 (AOR = 2.36 CI [1.26, 4.39]) times more associated with good hypoglycemia prevention practice than their counter part.

Being a government employee had a significant association with the prevention of hypoglycemia. Government employees were found 5.19 times higher more likely to have good hypoglycemia prevention practice than farmers (AOR = 5. 19, 95% CI [1.63, 16.58]).

Being divorced had negative statistical association with good hypoglycemia prevention practice. Those who were divorced had 87% less good hypoglycemia prevention practice than single participants (AOR = 0.13, 95% CI [0.32, 0.53]) (see Table 3).

## 4. Discussion

The study provided an insight into the level of hypoglycemia prevention practice and associated factors among patients with diabetes mellitus in public hospitals of the central zone, Tigray regional state, Ethiopia, 2018.

The magnitude of good hypoglycemia prevention practice among diabetes mellitus was found 63.2% (95% CI: 58.6, 68.8). This finding is considerably higher than the study finding documented in South Gondar, Northwest Ethiopia, which was found to be 21.4% [14]. This discrepancy could be due to sample size difference, educational status differences, and lack of awareness on the importance of hypoglycemia prevention practice. However, in a study conducted in Lebanon majority (96.1%) of diabetic patients managed a hypoglycemic episode adequately [18]. This discrepancy could be explained by the difference in the data collection tool.

Good knowledge on hypoglycemia was found significantly associated with good hypoglycemia prevention practice. The odds of knowledge was 10.34 times strongly associated with good hypoglycemia prevention practice This finding was supported by the study findings conducted in Benishangul Gomez Regional State Public Hospital, Nekemte Hospital, and Addis Ababa Hospital [19–21]. This association could reflect good knowledge that can enable participants how to prevent hypoglycemia and thus promotes patients' practical prevention of hypoglycemia.

Having a glucometer at home had statistically significant association with good hypoglycemia prevention practice. A comparable finding was also found in studies conducted in public hospitals of the central zone, Tigray [22], Mekelle and Ayder Referral Hospital [23], Benishangul Gomez Regional State Public Hospitals [19], and Addis Ababa Hospital [21]. This might be due to having a glucometer at home could reinforce patients to measure blood glucose level and apply recommended treatment changes immediately. Moreover, regular blood glucose level measurement might lead to lifestyle change that prevents hypoglycemia.

This study revealed that favorable attitude towards diabetes mellitus was found statistically associated with good hypoglycemia prevention practice. Participants who had favorable attitude towards diabetes mellitus were 2.36 times more likely to have good hypoglycemia prevention practice than to their counterparts. A comparable finding was obtained in the studies done in the public Hospital of the central zone of Tigray [22], Dilla University Referral Hospital [24], Nekemte Hospital [20], and Nigeria Hospital [25]. This might be due to having favorable attitude towards acute/chronic diseases such as diabetes mellitus is usually shown as an essential factor to create a healthy lifestyle change that prevents overall burden and complications of the health problem such as the hypoglycemia in diabetes mellitus.

This study showed that, government employees were found 5.19 times more likely to engage in good hypoglycemia prevention practice than farmers. This study finding is backed by the study conducted in Nekemte Referral Hospital in 2014 [26]. This similarity might be because of the educational status of the government employed participants are prone to media that lead to favorable attitude and good knowledge about hypoglycemia.

Divorced participants were found 87% less likely to have good hypoglycemia prevention practice. This could be defined by the experience of separation or divorce confers risk for poor health outcomes, including a 23% higher mortality rate [27].

## 5. Conclusion

Around two-thirds of the study participants were found to be good practice on prevention of hypoglycemia. Good knowledge and favorable attitude of diabetes mellitus, having a glucometer at home, governmental employee, and divorce of the study participants were found to be the predictors of good practice on prevention of hypoglycemia.

Therefore, healthcare personnel and the Ethiopian Diabetic Association should promote patients' attitude towards DM and knowledge on hypoglycemia by strengthening information, education, and communication program. Stakeholders should also try to provide glucometers to diabetic patients.

## Figures and Tables

**Table 1:** Sociodemographic profile of the participants on practice in prevention of hypoglycemia among patients with diabetes mellitus at public hospitals of the central zone, Tigray, Ethiopia 2018.

Variable	Category	Hypoglycemia prevention practice		Total
		Poor practice	Good practice	
Sex	Male	46 (16.9%)	97 (35.7%)	143 (52.6%)
	Female	54 (19.9%)	75 (27.6%)	129 (47.4%)

Age	≤34	29 (11.2%)	47 (18.1%)	76 (29.2%)
	35–64	56 (21.5%)	92 (35.4%)	148 (56.9%)
	≥65	10 (3.8%)	26 (10%)	36 (13.8%)

Residence	Urban	55 (20.2%)	118 (43.4%)	173 (63.6%)
	Rural	45 (16.5%)	54 (19.9%)	99 (36.4%)

Marital status	Married	50 (18.4%)	107 (39.3%)	157 (57.7)
	Divorced	32 (11.8%)	32 (11.8%)	64 (23.6)
	Widowed	11 (4%)	17 (6.3%)	28 (10.3)
	Single	7 (2.6%)	16 (5.9%)	23 (8.5)

Educational status	Cannot read and write	25 (9.2%)	35 (12.9%)	60 (22.1%)
	Can read and write	20 (7.4%)	52 (19.1%)	78 (28.7%)
	Primary school	20 (7.4%)	24 (8.8%)	44 (16.2%)
	Secondary school	26 (9.6%)	52 (19.1%)	78 (28.7%)
	College and above	15 (5.5%)	23 (8.5%)	38 (14%)

Occupation	Housewife	30 (11%)	40 (14.7%)	70 (25.7%)
	Daily worker	13 (4.8%)	29 (10.7%)	42 (15.4%)
	Self-employed	20 (7.4%)	44 (16.2%)	64 (23.5%)
	Governmental	14 (5.1%)	30 (11%)	44 (16.2%)
	Farmer	23 (8.5%)	29 (10.7%)	52(19.1%)

**Table 2:** Health profile, knowledge, and attitude towards hypoglycemia prevention practice among patients with diabetes mellitus at public hospitals, central zone, Tigray, Ethiopia, 2018.

Variable	Category	Poor practice	Good practice	Total
Type of DM	Type one	42 (15.4%)	71 (26.1%)	113 (41.5%)
	Type two	58 (21.3%)	101 (37.1%)	159 (58.5)

Diabetic complication	Yes	26 (9.2%)	52 (19.1%)	77 (28.3%)
	No	75 (27.6%)	120 (44.1%)	195 (71.7%)

Family history of DM	Yes	17 (6.3%)	26 (9.6%)	43 (15.8%)
	No	83 (30.5%)	146 (53.7%)	229 (84.1%)

Attending diabetic education	Yes	59 (21.7%)	120 (44.1%)	179 (65.8%)
	No	41 (15.1%)	52 (19.1%)	93 (34.2%)

Member of diabetic association	Yes	17 (6.3%)	40 (14.7%)	57 (21%)
	No	41 (15.1%)	79 (29%)	120 (44.1%)
	I did not know	42 (15.4)	53 (19.5)	95 (34.9)

Having a glucometer	Yes	8 (2.9%)	41 (15.1%)	49 (18%)
	No	92 (33.8%)	131 (48.2%)	223 (82%)

Knowing their current glucose level	Yes	54 (19.9%)	104 (38.2%)	158 (58.2%)
	No	46 (16.9%)	68 (25%)	114 (41.8%)

Attitude towards DM	Favorable	36 (13.2%)	96 (35.3%)	132 (48.5%)
	Unfavorable	64 (23.5%)	76 (27.9%)	140 (51.4%)

Knowledge towards hypoglycemia	Good	36 (13.2%)	143 (52.6%)	179 (65.8%)
	Poor	64 (23.5%)	29 (10.7%)	93 (33.8%)

**Table 3:** Bivariate and multivariable logistic regression analysis result of significant variables among patients with diabetes mellitus at public hospitals of the central zone of Tigray, Ethiopia in 2018.

Variable	Category	Hypoglycemia prevention practice		COR	AOR
		Good, *n* (%)	Poor, *n* (%)		
Marital status	Married	50 (18.4)	107 (39.3)	0.94 [0.36, 2.42]	0.57 [0.16, 1.96]
	Divorced	32 (11.8)	32 (11.8)	0.44 [0.16, 1.21]	0.13 [0.32, 0.53]^*∗*^
	Widowed	11 (4)	17 (6.3)	0.67 [0.21, 2.17]	0.34 [0.78, 1.49]
	Single	7 (2.6)	16 (5.9)	1	1

Occupation	Housewife	30 (11)	40 (14.7)	1.05 [0.51, 2.18]	0.72 [0.28, 1.78]
	Daily worker	13 (4.8)	29 (10.7)	1.76 [0.75, 4.15]	0.9 [0.31, 2.65]
	Self-employed	20 (7.4)	44 (16.2)	1.74 [0.82, 3.73]	1.76 [0.69, 4.52]
	Governmental	14 (5.1)	30 (11)	1.7 [0.73, 3.93]	5.19 [1.63, 16.58]^*∗*^
	Farmer	23 (8.5)	29 (10.7)	1	1

Diabetic education	Yes	59 (21.7)	120 (44.1)	1.6 [0.96, 2.68]	0.88 [0.44, 1.75]
	No	41 (15.1)	52 (19.1)	1	1

Having glucometer	Yes	8 (2.9)	41 (15.1)	3.59 [1.61, 8.03]	3.02 [1.12, 8.12]^*∗*^
	No	92 (33.8)	131 (48.2)	1	1

Attitude towards DM	Favorable	36 (13.2)	96 (35.3)	2.25 [1.35, 3.73]	2.36 [1.26, 4.39]^*∗*^
	Unfavorable	64 (23.5)	76 (27.9)	1	1

Knowledge towards hypoglycemia	Good	36 (13.2)	143 (52.6)	8.76 [4.95, 15.52]	10.34 [5.41, 19.89]^*∗*^
	Poor	64 (23.5)	29 (10.7)	1	1

^*∗*^Those variables significantly associated with the outcome variable at *p* value < 0.05.

## Data Availability

The data sets used and analyzed during the current study could be made available upon reasonable request to the corresponding author.

## Abbreviations

AOR: Adjusted odds ratio
COR: Crude odds ratio
DM: Diabetes mellitus
FBS: Fasting blood sugar
IEC: Information education and communication
IRB: Institutional review board
MMS: Modified Morse Scale
SMBG: Self-monitoring blood glucose.
